# A cohort study to identify and evaluate concussion risk factors across multiple injury settings: findings from the CARE Consortium

**DOI:** 10.1186/s40621-018-0178-3

**Published:** 2019-01-14

**Authors:** Kathryn L. Van Pelt, Dain Allred, Kenneth L. Cameron, Darren E. Campbell, Christopher J. D’Lauro, Xuming He, Megan N. Houston, Brian R. Johnson, Tim F. Kelly, Gerald McGinty, Sean Meehan, Patrick G. O’Donnell, Karen Y. Peck, Steven J. Svoboda, Paul Pasquina, Thomas McAllister, Michael McCrea, Steven P. Broglio

**Affiliations:** 1 NeuroTrauma Research Laboratory, University of Michigan, 401 Washtenaw Ave, Ann Arbor, MI 48104 USA; 2 United States Air Force Academy, 2355 Faculty Drive, Suite 1N207, U.S. Air Force Academy, CO 80840 USA; 3 John A. Feagin Jr. Sports Medicine Fellowship, Keller Army Community Hospital, 900 Washington Rd, West Point, NY 10996 USA; 4 Department of Statistics, University of Michigan, 1085 South University, Ann Arbor, MI 48109 USA; 5 Department of Intercollegiate Athletics, United States Military Academy, 639 Howard Road, West Point, NY 10996 USA; 6 School of Kinesiology, University of Michigan, 401 Washtenaw Ave, Ann Arbor, MI 48104 USA; 7 United States Coast Guard Academy, 31 Mohegan Ave Pkwy, New London, CT 06320 USA; 8 Department of Rehabilitation Medicine, Walter Reed National Military Medical Center, Uniformed Services University of the Health Sciences, 4301 Jones Bridge Road, Bethesda, MD 20814 USA; 9 Indiana University School of Medicine, Goodman Hall, 355 W. 16th St., Suite 4800, Indianapolis, IN 46202 USA; 10 Medical College of Wisconsin, Department of Neurosurgery, 8701 Watertown Plank Road, Milwaukee, WI 53226 USA; 11 MedStar, 1120 20th Street NW, Washington DC, 20036 USA

**Keywords:** Traumatic brain injury, Risk, Injury, Epidemiology

## Abstract

**Background:**

Concussion, or mild traumatic brain injury, is a major public health concern affecting 42 million individuals globally each year. However, little is known regarding concussion risk factors across all concussion settings as most concussion research has focused on only sport-related or military-related concussive injuries.

**Methods:**

The current study is part of the Concussion, Assessment, Research, and Education (CARE) Consortium, a multi-site investigation on the natural history of concussion. Cadets at three participating service academies completed annual baseline assessments, which included demographics, medical history, and concussion history, along with the Sport Concussion Assessment Tool (SCAT) symptom checklist and Brief Symptom Inventory (BSI-18). Clinical and research staff recorded the date and injury setting at time of concussion. Generalized mixed models estimated concussion risk with service academy as a random effect. Since concussion was a rare event, the odds ratios were assumed to approximate relative risk.

**Results:**

Beginning in 2014, 10,604 (*n* = 2421, 22.83% female) cadets enrolled over 3 years. A total of 738 (6.96%) cadets experienced a concussion, 301 (2.84%) concussed cadets were female. Female sex and previous concussion were the most consistent estimators of concussion risk across all concussion settings. Compared to males, females had 2.02 (95% CI: 1.70–2.40) times the risk of a concussion regardless of injury setting, and greater relative risk when the concussion occurred during sport (Odds Ratio (OR): 1.38 95% CI: 1.07–1.78). Previous concussion was associated with 1.98 (95% CI: 1.65–2.37) times increased risk for any incident concussion, and the magnitude was relatively stable across all concussion settings (OR: 1.73 to 2.01). Freshman status was also associated with increased overall concussion risk, but was driven by increased risk for academy training-related concussions (OR: 8.17 95% CI: 5.87–11.37). Medical history of headaches in the past 3 months, diagnosed ADD/ADHD, and BSI-18 Somatization symptoms increased overall concussion risk.

**Conclusions:**

Various demographic and medical history factors are associated with increased concussion risk. While certain factors (e.g. sex and previous concussion) are consistently associated with increased concussion risk, regardless of concussion injury setting, other factors significantly influence concussion risk within specific injury settings. Further research is required to determine whether these risk factors may aid in concussion risk reduction or prevention.

**Electronic supplementary material:**

The online version of this article (10.1186/s40621-018-0178-3) contains supplementary material, which is available to authorized users.

## Introduction

Concussion, or mild traumatic brain injury (mTBI), is a prevalent injury among athletes and military service members. An estimated 1.6–3.8 million sport and recreation-related concussions occur annually (Langlois et al., 2006). Concussion is deemed the signature injury of recent wars, affecting 14.9%–22.8% of service members (Wilk et al., 2010; Hoge et al., 2008; Terrio et al., 2009). High concussion prevalence among athletes and service members is a growing public health concern.

Concussion epidemiological studies have studied athletes (Zuckerman et al., 2015), service members (Helmick et al., 2015; Cameron et al., 2012), and emergency department admissions (Bazarian et al., 2005) to better understand concussion burden within each setting. However, to date, such studies have characterized concussion risk factors separately within each subpopulation and typically only related to a particular mechanism (e.g. sport-related). However, athletes and service members are not immune to concussions outside of the sport arena or combat. For example, 80% of concussions sustained by service members occurred outside of active war zones (Cameron et al., 2012), likely due to a combination of sport, recreation, military training (e.g. boxing) related activities, and non-recreation activities (e.g. motor vehicle crash, fall, etc.). Furthermore, to evaluate multiple concussion risk factors requires a large sample size. Previous studies may have been underpowered to assess multiple risk factors simultaneously, or had too few females to evaluate sex-specific risk factors. Elucidating individual characteristics that increase concussion risk is essential to fully characterizing concussion and its burden and provides a pathway to mitigating risk.

To reduce concussive injuries, recent research focused on identifying individual-level concussion risk factors. Female high school and college athletes have 1.5 to 2.1 times greater concussion risk than their male peers (Lincoln et al., 2011; Covassin et al., 2003). A combination of physical, psychological, and physiological differences are possible reasons for increase female concussion risk (Dick, 2009). Unlike sport-related concussions, little is known to date about factors that impact female concussion risk in the military. However, in light of evolving female combat roles, studies investigating female concussion risk among service members are needed. We hypothesized that female cadet athletes will have increased sport-related concussion risk relative to males (Lincoln et al., 2011; Covassin et al., 2003). Relative to females, males are at increased risk for military-related concussions (Hoge et al., 2008) . We hypothesized that males will have greater training-related and free time-related concussion risk and rates. Previous concussion and increased baseline psychological symptoms are also expected to increase concussion risk.

In addition to sex, medical comorbidities such as learning disorders, migraine headaches (Kutcher & Eckner, 2010), previous concussion (Abrahams et al., 2014), and pre-morbid symptoms (Schneider et al., 2013) all influence concussion risk. Relative to other medical comorbidities, having a previous concussion had the strongest evidence for increasing subsequent concussion risk (Abrahams et al., 2014). A systematic review of concussion risk factors reported increased concussion risk in athletes with a previous concussion in 10 of 13 studies (Abrahams et al., 2014). The association between previous concussion and incident concussion was observed in a variety of sports (Abrahams et al., 2014). However, nine of the ten studies enrolled all male or 99% male participants. While previous concussion appears to increase incident concussion risk, future research should test this relationship across sexes.

Baseline or pre-concussion symptoms, such as headache, have been associated with increased concussion risk in youth ice hockey players (Schneider et al., 2013). Baseline symptoms also influence post-concussion symptoms, whereby higher baseline somatization symptoms increase concussion-related symptom burden or reporting (Nelson et al., 2016). Approximately 85% of clinicians use a concussion symptom checklist to diagnose a concussion, more than any other concussion evaluation tool (Baugh et al., 2016). Given the clinician’s reliance on concussion symptoms for diagnosis, increased symptom burden or reporting due to greater baseline symptom may increase concussion diagnosis likelihood. Thus, increased pre-concussion somatization or concussion symptoms may be associated with increased concussion risk due to physiological mechanisms or increased diagnosis.

Psychological symptoms and disorders, such as anxiety and depression, also are hypothesized to increase concussion risk (Kutcher & Eckner, 2010). A recent study of collegiate athletes found that increased baseline anxiety symptoms increased the likelihood of any injury setting in the following athletic-season for both sexes (Li et al., 2017). However, elevated depressive symptoms only increased subsequent injury risk for males (Li et al., 2017). Since the recent study did not distinguish between concussive and non-concussive injuries (e.g. ankle sprain), it is unclear whether increased psychological symptoms have a specific influence on concussion likelihood. Consequently, the current study sought to determine the role of psychological symptoms on subsequent concussion risk. We hypothesized that increased pre-injury concussion, somatization, anxiety, and depression symptoms would increase the risk for subsequent concussion.

The current study aimed to address whether a variety of factors influence concussion risk across different injury settings. These factors span demographic (e.g. sex), medical and well-being (e.g. previous concussion, psychological symptoms), and environment (e.g. playing varsity level sport). Risk factors characterized across any concussion along with sport-related, academy training-related, and free time-related settings will yield a more comprehensive understanding of concussion incidence given various individual and environmental risk factors.

## Methods

### Study design

The current study is part of a larger joint effort by the U.S. Department of Defense (DoD) and National Collegiate Athletic Association (NCAA). This partnership funded the Concussion, Assessment, Research and Education (CARE) Consortium, a multi-site investigation on the natural history of concussion. A detailed description of the CARE Consortium has been published previously, along with detailed descriptions of the measures and variables evaluated as risk factors (Broglio et al., 2017). Site-level Institutional Review Board (IRB) and DoD Human Research Protections Office (HRPO) approval was obtained (Broglio et al., 2017) and all participants provided consent before testing.

Concussion risk factors were evaluated among service academy members (cadets) at the United States Service Academies. Cadets at the United States Service Academies are unlike active duty service members in that there is a larger female population, they have different social, economic, and education characteristics than enlisted service members, and their academy military training activities do not include exposure to combat (O'Connor et al., 2018). Cadets are a unique population, enabling researchers to evaluate multiple concussion risk factors across collective and separate injury settings (e.g. sport-related, academy training-related, and free time-related) within the same population.

Between 2014 and 2017 all cadets at the participating military service academies were eligible to enroll. Each cadet is required to participate in a sport, thus cadets participated at either varsity (NCAA sports), club, or intramural levels. Before each academic year, participants completed a baseline assessment of self-reported demographic information, medical history, sport participation history, and socio-economic status questions (Broglio et al., 2017).

Incident concussions were defined by evidence-based guidelines, based on a systematic review of concussion indicators (Carney et al., 2014). The results of this review define a concussion as:

“a change in brain function following a force to the head, which may be accompanied by temporary loss of consciousness, but is identified in awake individuals with measures of neurologic and cognitive dysfunction” (Carney et al., 2014).

Clinicians at each site were provided the concussion definition and were asked to diagnose concussions based on their clinical judgment and the definition characterized by Carney and colleagues (Carney et al., 2014). Concussion evaluations were conducted if a cadet self-reported concussion-like symptoms; if a fellow cadet reported concern, or if the clinician, researcher, or staff observed an event with a significant head impact. The cadet would then meet with the clinician to complete a post-injury assessment including measures of concussion symptoms, balance, and cognition. Based on the event description and clinical measures, the clinician would make their diagnosis. After a diagnosed concussion, research staff recorded the injury’s characteristics, including the date and time of injury and the injury setting. Since all cadets must participate in both sport and academy training activities (O'Connor et al., 2018) all cadets were at risk for concussions during these activities.

Concussion setting was categorized as sport (varsity, club, intramural level), academy training (physical education, military training, or boxing), or free time-related. Free time-related concussions were defined as those not occurring during any structured Service Academy activity. For cadets sustaining multiple incident concussions during the study period, each concussion was coded. For example, a cadet sustaining a concussion during football and a second concussion during an academy training exercise would have one positive indicator for sport-related concussion and one for academy training-related concussion. Alternatively, if a football player sustained two concussions during football both concussions would be recorded and coded as sport-related injuries. Concussion risk was estimated across all concussions and for each concussion setting: sport-related, academy training-related, free time-related.

Because cadets completed annual assessments, there were multiple assessments available for cadets who participated in the CARE study for more than one year. For those cadets who did not sustain an incident concussion, their most recent assessment relative to data extraction (June 2017) was utilized. For those cadets who sustained a concussion, the assessment that preceded their first concussion was used.

Each annual assessment asked cadets to complete or update health and environment information. Demographic information that was completed at the first assessment (i.e. sex) was assumed to stay constant throughout the study period and was not surveyed in subsequent years. Medical history information was updated annually and included concussion history, headache, migraine, ADD/ADHD, and symptom evaluations. The annual assessment also included concussion and psychological symptom evaluations to obtain the cadet’s typical symptom burden. The Sport Concussion Assessment Tool (SCAT) symptom inventory evaluated concussion symptoms (McCrory et al., 2013). The SCAT measured the number and severity of concussion related symptoms, including headache, nausea, and dizziness. Psychological variables were measured using the Brief Symptom Inventory-18 (BSI-18) (Derogatis, 2001). The BSI-18 measures the severity of anxiety, depression, and somatization-related symptoms.

Cadets are required to participate in an athletic activity. These athletic activities span three sport levels: varsity, club, and intramural. varsityCadet’s sport level was defined based on the sport indicated in the first evaluation. Sport level categories are assumed to be mutually exclusive for the current analysis, however, it is possible cadets switched sport levels in subsequent years. Varsity level athletes were defined as cadets competing in NCAA sanctioned sports. Varsity level status was also sub-categorized based on the contact sport levels outlined by Rice (Rice, 2008). VarsityClub level athletes compete competitively against other schools in non-NCAA sports while intramural athletes compete in sports within their academy. Further details regarding the specific sports in each sport level has been described previously (O'Connor et al., 2018).

### Statistical analysis

Univariate tests first assessed for associations between risk factors, sex, and incident concussion in order to identify possible sex-dependent risk factors. Table 1 summarizes relationships between proposed risk factors and concussion. Incident concussion was the presence (yes/no) of any concussion across four injury settings: all settings, sport-related, academy-related, and free time-related. Chi-squared tests assessed for associations between nominal variables (sport level, contact level, freshman status, site, previous concussion, headaches, migraine, ADD/ADHD, diagnosed depression). Independent sample t-tests assessed for differences in continuous variables (Brief Sensation Seeking Scale (BSSS)). The Satterthwaite t-test was used if the variances across groups were unequal. Furthermore, for non-normally distributed variables the non-parametric Wilcoxon test was used (SCAT and BSI-18). Due to the large sample size in the study, effect sizes were estimated for each significant finding. Phi (Φ) was the effect size estimated for categorical variables with more than two levels. Small, medium, and large effect sizes were defined as 0.1, 0.3, and 0.5, respectively (Cohen, 1988). For continuous variables and nominal variables with only two levels, Cohen’s d was calculated. Odds ratios were converted to Cohen’s d effect sizes using the formula from Chinn and colleagues (Chinn, 2000). Effect size magnitudes for Cohen’s d were defined as 0.3, 0.5, and 0.8 for small, medium, and large magnitudes (Cohen, 1988). Finally, the effect size r was estimated when non-parametric tests were used.

**Table 1:** Univariate associations between proposed risk factors and concussion

	Any Concussion % (n) Mean (SD) Median [IQR]	Sport % (n) Mean (SD) Median [IQR]	Academy Training % (n) Mean (SD) Median [IQR]	Free Time % (n) Mean (SD) Median [IQR]
Sport Level				
Varsity	9.14% (268)***	5.81% (169)***	1.82% (53)**	0.96% (28)
Club	7.09% (132)	4.25% (79)	1.99% (37)	0.97% (18)
Intramural	5.65% (301)	1.23% (65)	2.85% (151)	1.09% (58)
Sex				
Female	11.30% (227)***	4.36% (105)***	3.91% (94)***	2.24% (54)***
Male	5.84% (460)	2.83% (217)	1.95% (150)	0.81% (62)
Contact Level^a^				
Contact		8.12% (144)***		
Limited-Contact		3.83% (14)		
Non-Contact		1.55% (11)		
Freshman				
Yes	13.38% (369)***	5.02% (137)***	6.77% (185)***	1.43% (39)
No	4.82% (355)	2.51% (184)	0.79% (58)	1.05% (39)
Site				
1	6.15% (280)***	3.31% (150)**	1.66% (75)***	0.95% (43)
2	9.00% (421)	3.45% (160)	3.60% (167)	1.40% (65)
3	2.71% (25)	1.30% (12)	0.22% (2)	0.87% (8)
Previous Concussion				
Yes	10.72% (215)***	5.39% (107)***	3.37% (67)**	1.46% (29)*
No	5.92% (475)	2.50% (200)	2.14% (171)	0.94% (75)
Headaches in past 3 months				
Yes	11.18% (251)***	4.64% (103)***	4.64% (103)***	1.31% (29)
No	5.67% (438)	2.64% (203)	1.77% (103)	0.96% (74)
Diagnosed Migraine Headache				
Yes	11.37% (29)**	5.16% (13)	3.97% (10)	1.98% (5)
No	6.77% (659)	3.02% (292)	2.37% (229)	1.00% (97)
Diagnosed ADD/ADHD				
Yes	12.95% (18)**	3.65% (5)	7.30% (10)**	1.46% (2)
No	6.73% (661)	3.06% (299)	2.36% (230)	1.02% (100)
Diagnosed Depression				
Yes	13.46% (14)**	3.88% (4)	5.83% (6)*	2.91% (3)
No	6.83% (676)	3.07% (302)	2.38% (234)	1.01% (99)
Brief Sensation Seeking Scale	3.37 (0.66)	3.38 (0.69)	3.35 (0.57)	3.46 (0.76)
Baseline SCAT Symptoms				
Number	2.00 [0.00–6.00]***	2.00 [0.00–5.00]***	4.00 [1.00–9.50]***	2.00 [0.00–5.00]
Severity	4.00 [0.00–11.00]***	2.00 [0.00–9.00]***	7.00 [1.50–18.00]***	3.00 [0.00–7.00]*
Baseline BSI Total	39.00 [36.00–48.00]***	36.00 [36.00–47.00]*	45.00 [36.00–52.00]***	36.00 [36.00–45.00]
Somatization	42.00 [42.00–50.00]***	42.00 [42.00–48.00]**	48.00 [42.00–58.00]***	42.00 [41.00–48.00]
Depression	42.00 [42.00–45.00]	42.00 [42.00–45.00]	42.00 [42.00–48.00]***	42.00 [40.00–45.00]*
Anxiety	39.00 [39.00–47.00]	39.00 [39.00–47.00]	39.00 [39.00–48.00]***	39.00 [38.00–45.00]

* *p* < 0.05; ** *p* < 0.01; *** *p* < 0.001

^a^ Contact level only evaluated for varsity cadets

Full tables available in additional file

Percentages and sample size (% (n)); mean and standard deviation (mean (SD)); median and IQR (median [IQR])

Generalized mixed models were used to determine concussion risk factors across multiple activities. Because concussion was a rare event, occurring in less than 10% of cadets, odds ratios were assumed to approximate relative risk (Rothman, 2012). Accounting for clustering effect of Service Academy a random effect of academy was included in each model. Each concussion model first assessed the random effect of academy in an empty model without any covariates. For each concussion setting, the random effect of academy was significant and thus was included in each model.

The maximum likelihood method, Laplace, was used to identify a final model. Each predictor was added to the model individually. The only exception was the BSI-18 sub-scores which were added simultaneously. For each variable to remain in the model, it must be significant, the likelihood ratio test significant, and the Bayesian information criterion (BIC) value lower than the reference model. After a final model was selected, a generalized mixed model was fit using the pseudo-likelihood method to confirm the significant predictors. Model selection tables are included in the (Additional file 1: Tables S3, S6, S9, S12, S14, S16, S18, S19). Univariate associations are also provided in the additional file (Additional file 1: Tables S2, S5, S8, S11). Additional file tables describing the multivariate model results are specified in the text and summarized in Fig. 2. Full results tables are provided in the additional file (Additional file 1: S4, S7, S10, S14, S15, S17, S19). All analyses were completed using SAS version 9.4 (Cary, NC).

## Results

### Cadet characteristics

Starting in August 2014 and ending June 2017, three Service Academies contributed data from 10,604 (*n* = 2421, 22.83% female) cadets, with 4581 (43.20; *n* = 934, 8.81% female) at site 1, 5066 (47.78%; *n* = 1239, 11.68% female) at site 2, and 953 (8.99%; *n* = 348, 3.28% female) at site 3.

### Concussion characteristics

Out of 800 injuries during the study period an injury setting designation was not assigned in 81 cases. There was no association between sex or site with the likelihood of missing injury activity data. School year was significantly associated with missing injury setting data (*X*^2^ (2) = 178.07; *p* < 0.001), the greatest proportion of which occurring during the 2014–2015 academic year, with decreasing missingness each subsequent study year. The larger proportion of missing data in the 2014–2015 academic year was attributed to the rapid start of the study. Also, sport level (varsity, club, intramural) was associated with missing injury setting data (*X*^2^ (3) = 41.98; p < 0.001). Club sport level was less likely to have missing data than varsity (*X*^*2*^ (1) = 6.25; *p* = 0.01) or intramural (*X*^*2*^ (1) = 8.86; *p* = 0.003). There was no difference between varsity and intramural. Injuries among cadets who skipped the sport level question were most likely to have missing data for injury setting.

A total of 800 (*n* = 301 female) concussions occurred among 738 (6.96%) cadets. There were 679 (*n* = 253, 37.26% female), 56 (*n* = 24, 42.86% female), and 3 (*n* = 0 female) cadets who experienced one, two, or three concussions respectively during the study period. There were 33 cadets (*n* = 11, 33.33% female) that had a repeat concussion within the same academic year (66 injuries total injuries). Of the same-season injuries, three occurred within fourteen days of the first injury, and of these, two occurred within seven days of the first injury.

**Fig. 1:**
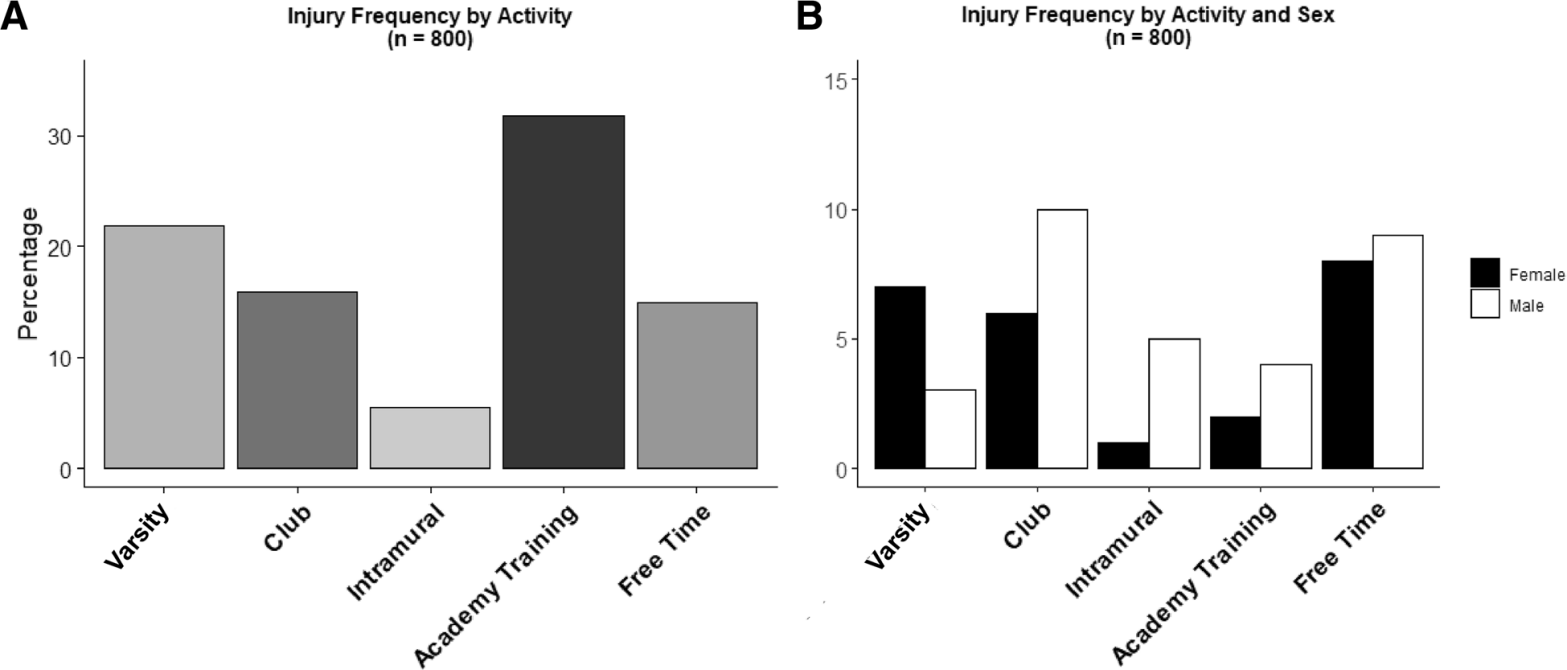
**a.** Percentage of concussive injuries by injury setting. The majority of concussive injuries occured during sport (varsity, club, or intramural) followed by Academy Training and Free Time. **b.** Percentage of concussive injuries by injury setting and sex. For both female and males, the majority of concussive injuries occured during sport (varsity, club, or intramural level)

Among all concussions, injuries occurred during the following settings: 24.31% (*n* = 175) varsity sports, 17.66% (*n* = 127) club sports, 6.12% (*n* = 44) intramural sports, 22.81% (*n* = 164) physical education class, 12.52% (*n* = 90) academy training, and 16.55% (*n* = 119) during free time (Fig. 1a). Injury setting across sexes are presented in Fig. 1b.

### Univariate asssesment of concussion risk

Univariate concussion risk factors were estimated for any concussion, sport-related, academy training-related, and free-time related concussions. The univariate assessment of concussion risk factors is summarized in Table 1. For any incident concussion, sport level, sex, freshman status, previous concussion, headache, diagnosed migraine, diagnosed ADD/ADHD, diagnosed depression, SCAT symptoms, and BSI somatization score were all significantly associated with any incident concussion (all p’s < 0.01; Additional file 1: Table S2). However, only freshman status had a medium or larger effect size, with over three times the risk of any concussion compared to non-freshman (OR = 3.05; 95% CI: 2.62–3.56) (Table 1). 

Sport-related concussions were significantly associated with sport level, sex, freshman status, previous concussion, headaches, and BSI somatization scores (all p’s < 0.05; Additional file 1: Table S5). Contact sport level had the largest effect size (Φ = 1.22), with all other predictors having small effect sizes. Relative to limited-contact sport cadets, contact sport cadets had 2.22 greater risk for any sport-related concussion (OR = 2.22; 95% CI: 1.30–4.03). Risk for sport-related concussion was greater for both contact (OR = 5.60; 95% CI: 3.11–10.92) and limited-contact sport cadets (OR = 2.52; 95% CI: 1.12–5.76) when compared to non-contact sport cadets.

Academy training-related concussions were associated with sport level, sex, freshman status, previous concussion, headache, diagnosed ADD/ADHD, diagnosed depression, SCAT symptoms (number and severity) at baseline, and all BSI symptom (all *p* < 0.05; Additional file 1: Table S8). Freshman status and medical comorbidities (headache, diagnosed ADD/ADHD, and diagnosed depression) had medium to large effect sizes. Freshman had more than a nine-fold increased risk for incident academy training-related concussion relative to non-freshman (OR = 9.11; 95% CI: 6.76–12.27). Cadets who reported having headaches in the three months before their baseline had 2.70 times increased training-related concussion risk than those who did not have headaches (OR = 2.70; 95% CI: 2.08–3.51). Similarly, cadets with diagnosed ADD/ADHD or depression had increased risk of academy training-related concussions (Additional file 1: Table S8).

Sex, previous concussion, and BSI depression scores were significantly associated with free time concussions (all p’s < 0.05; Additional file 1: Table S11). However, only sex had at least a medium effect size. Females had 2.82 times the increased risk for free time concussions compared to males (OR = 2.82; 95% CI: 1.95–4.07).

Univariate tests evaluated sex as a possible moderator. Thus, concussion predictors were assessed for their association with sex (Additional file 1: Table S1). All variables were significantly associated with sex (all p’s < 0.05). The largest effect sizes were observed with sport level, contact level among varsity cadets, diagnosed depression, and BSI scores. Interactions between sex and these variables were assessed in the mixed models.

### Multivariate assessment of concussion risk

Service Academy was a significant random effect for each mixed model. Each subsequent model included site as a random effect. All models are included in the additional file and the final models are interpreted below.

**Fig. 2:**
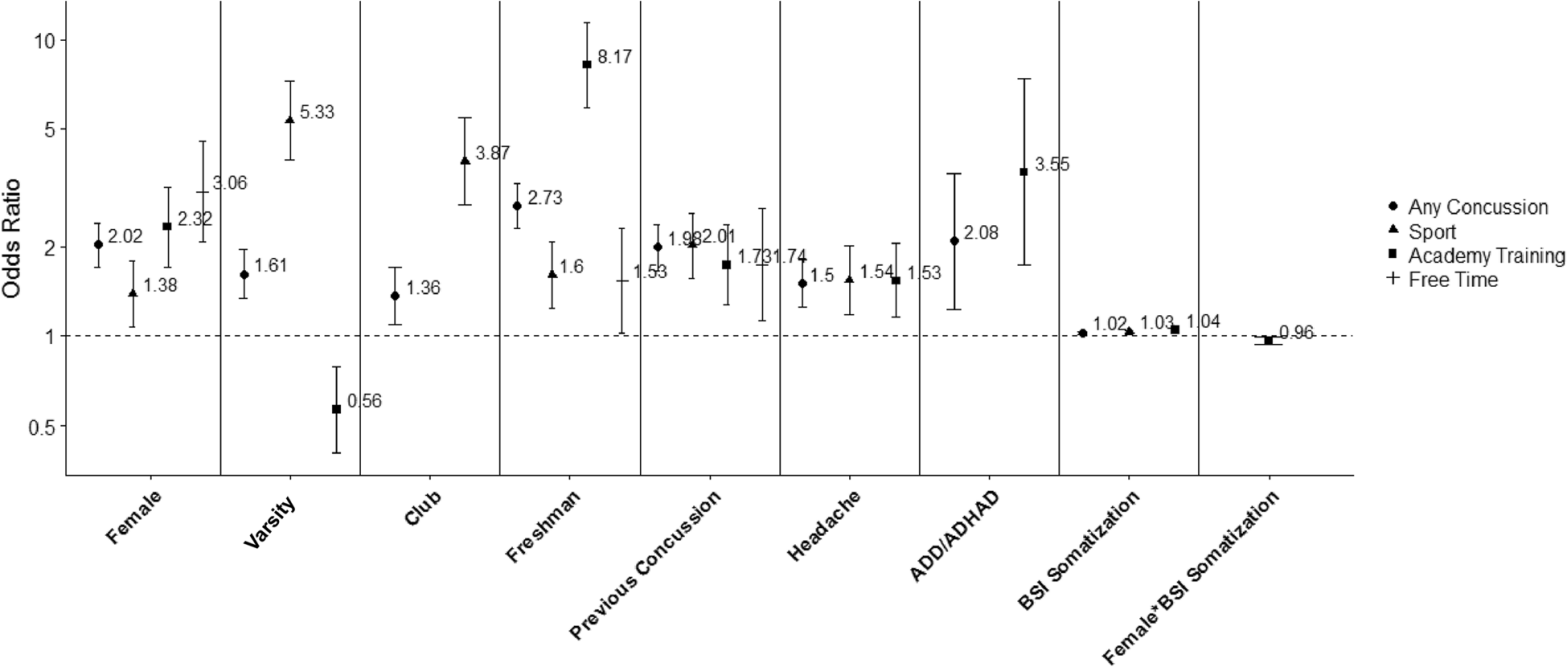
Summary of Concussion Risk Factors by Concussion Setting Across All Cadets: Summary of Multivariate Models

Females, freshman status, and varsity and club cadets were characteristics associated with increased risk for any concussion (Fig. 2, Additional file 1: Table S4). Additionally, medical history including previous concussion, headache in the past 3 months, and diagnosed ADD/ADHD all increased concussion risk. Finally, increasing BSI somatization symptom scores were associated with increased concussion risk (Fig. 2, Additional file 1: Table S4). Each factor was significant after controlling for all other covariates. There were no significant interactions between sport level, diagnosed depression or BSI symptom scores with sex (all *p* > 0.05).

As observed with concussions of any setting, female sex, varsity and club status, and freshman status were characteristics associated with increased risk for sport-related concussion (Fig. 2, Additional file 1: Table S7). Varsity cadets had the greatest risk for sport-related concussion compared to club (OR = 1.38; 95% CI: 1.03–1.83) and intramural cadets (OR = 5.33; 95% CI: 3.93–7.22). Previous concussion and headache were associated with two and 1.5 times greater risk for sport-related concussion, respectively (Fig. 2, Additional file 1: Table S7). Finally, higher BSI somatization symptom scores were associated with increased sport-related concussion risk (Fig. 2, Additional file 1: Table S7).

Among academy training-related concussions, sex significantly interacted with BSI somatization symptoms. With increasing BSI somatization symptoms, the risk for academy training-related concussion increased, but at a greater rate for males compared to females (Fig. 2, Additional file 1: Table S10). Varsity cadets had a 44% lower risk for academy training-related concussions compared to intramural cadets (OR = 0.56; 95% CI: 0.40–0.78). There were no significant differences between varsity and club, or club and intramural cadets. Freshman had eight times greater risk for an academy training-related concussion compared to non-freshman (OR = 8.17; 95% CI: 5.87–11.37). Previous concussion, headache, and diagnosed ADD/ADHD also significantly increased the risk of an academy-training-related concussion (Fig. 2, Additional file 1: Table S10).

Risk for free time-related concussions was significantly estimated by sex, freshman status, and previous concussion. Females, freshman, and cadets with a prior concussion were all at significantly increased risk for a free time-related concussion (Fig. 2, Additional file 1: Table S13).

Within varsity athlete cadets there was a significant sex and BSI anxiety symptom score interaction. With increasing BSI anxiety symptom scores, concussion risk increased at a greater rate for males than females (Additional file 1: Table S15). Contact sport cadets were at greater risk for any concussion compared with non-contact cadets (OR = 2.43; 95% CI: 1.60–3.68). There was no significant difference in risk between contact and limited-contact or limited-contact and non-contact cadets (both p’s > 0.05). As observed within the entire cadet sample, varsity females, freshman, cadets with a previous concussion, or cadets with headaches in the 3 months prior to baseline were all at increased risk for concussion (Additional file 1: Table S15).

Within varsity-sport injuries, females had 1.71 times the risk for a varsity sport concussion compared to males (Additional file 1: Table S17). Contact and limited-contact sport cadets had a greater risk of a sport-related concussion than non-contact sport cadets. Contact sport cadets had 2.15 times greater risk than limited-contact cadets for sport-related concussion (95% CI: 1.18–3.92). Previous concussion and increasing BSI somatization symptom scores also increased sport-related concussion risk (Additional file 1: Table S17).

Varsity cadets who were female (OR = 1.96; 95% CI: 1.08–3.56), freshman (OR = 15.82; 95% CI: 6.70–37.38), and who had a previous concussion (OR = 1.93; 95% CI: 1.06–3.16) had increased risk for an academy training-related concussion. For free time-related concussions, sex was the only significant predictor. Females had 2.89 (95% CI: 1.35–6.19) times greater risk for a free time-related concussion than males.

## Discussion

The objective of the current study was to determine concussion risk factors among United States Service Academies cadets across multiple injury settings. Different risk factors were observed for each concussion setting, highlighting specific at-risk populations within each setting. New findings revealed that varsity athlete cadets and increasing somatization symptoms were associated with increased concussion risk.

The large sample size and prospective study design powered the current study to assess how risk factors change based on injury setting. Rather than separate studies for each setting, the current study was able to assess risk factors for various concussion etiologies with the same participant sample. Future studies should further examine male and female differences to determine whether civilian females also experience greater concussion risk across multiple injury settings.

### Concussion risk factors

Female sex, freshman status, previous concussion, and headache history were the most consistent concussion risk factors captured at the baseline assessment. However, the strength of these associations varied by concussion setting. For example, females had the greatest risk and strongest effect for concussions of any setting and free time-related concussions while freshman status had the strongest effect on academy training-related injuries. Sport level had the greatest effect on sport-related concussions, while contact sport level had the strongest association for varsity sport-related injuries.

Females had increased overall, sport-related, academy training-related, and free time-related concussion risk, which is similar to previous findings within high school and collegiate athletes showing females have higher rates of sport-related concussions (Lincoln et al., 2011; Covassin et al., 2003; Abrahams et al., 2014). Outside of athletics, males have greater risk for military-related concussion (Hoge et al., 2008) and all traumatic brain injury (Bazarian et al., 2005; Cassidy et al., 2004). However, studies investigating concussion incidence within service members have limited their investigation to active-duty concussions and enrolled few females (Wilk et al., 2010; Hoge et al., 2008). Unlike previous studies of service members sustaining active-duty concussions (Hoge et al., 2008; Cameron et al., 2012) or emergency department admission studies (Bazarian et al., 2005; Cassidy et al., 2004), current results demonstrate that female cadets had increased risk for academy training-related and free time-related concussions. Thus, greater concussion risk among females is not limited to sports participation.

While the current results conflict with studies from military and emergency department epidemiological studies of concussion, there is biological plausibility why females may be at greater concussion risk. Lower neck strength among females relative to males is proposed to reduce the bracing capacities of female athletes, increasing their risk for concussion (Eckner et al., 2014; Tierney et al., 2005). Increased concussion rates may also be due to increased reporting among females. Females endorse more concussion-related symptoms (Covassin et al., 2012) and are more likely to report an injury (Wallace et al., 2017). Hormonal differences may also predispose females to concussions. Human studies have observed worse recovery when injured during the luteal phase of the menstrual cycle (Wunderle et al., 2014). Thus, it is unclear if females sustain more concussions due to physical, physiological or psychological mechanisms, of if reported concussion risk is artificially inflated due to reporting differences.

Previous concussion, headaches in the previous three months, and diagnosed ADD/ADHD disorders all increased concussion risk and replicated findings from the sports medicine literature (Kutcher & Eckner, 2010; Abrahams et al., 2014). Furthermore, results from the current study furthered the understanding of concussion risk by demonstrating previous concussion, headache, and diagnosed ADD/ADHD disorders all occurred before the incident concussion. Establishing temporality of pre-concussion medical diagnoses provided supporting causal evidence that previous concussion, headaches, and ADD/ADHD increase concussion risk.

Pre-existing headaches and ADD/ADHD diagnoses have not been assessed for incident concussion risk among service members or cadets. Headache symptoms and ADD/ADHD diagnoses have primarily been an outcome measure after TBI (Miller et al., 2015; Wells, 2010). In the military, screening for post-deployment TBI began in 2008, and focused on a service member’s most recent deployment (Iverson et al., 2009). Thus, establishing a TBI history was difficult, and to our knowledge, no study has examined the relationship between previous medical history and incident concussion among male and female service members.

Increased baseline anxiety, depression, somatization and concussion symptoms were hypothesized to increase cadet’s incident concussion risk. However, baseline depression symptoms did not influence subsequent concussion risk. There was a significant sex by anxiety symptom interaction for any concussion among varsity athlete cadets. In support of the hypothesis, concussion reporting increased with increasing baseline anxiety symptom scores, which has also been linked to risk for any injury (Li et al., 2017). The current study utilized the BSI-18 to assess psychological symptoms while Li and colleagues (Li et al., 2017) used the trait portion of the State-Trait Anxiety Inventory (Spielberger, 1989) and the Center for Epidemiologic Studies Depression Scale (Radloff, 1977). Additionally, rather than using the symptom scores as continuous variables, Li and colleagues (Li et al., 2017) set cutoffs to categorize participants as having elevated anxiety and depression symptoms (Li et al., 2017). While anxiety symptoms influence both general injury risk (Li et al., 2017) and concussion risk, depression symptoms only appear to be associated with general injury risk (Li et al., 2017), not concussion risk. Supporting the lack of association between depression and concussion risk the current study also observed that baseline depression symptoms did not influence subsequent concussion risk.

Finally, compared to the previous study by Li and colleagues (Li et al., 2017), somatization symptom scores were included in the current analysis. Somatization symptoms were found to significantly estimate any concussion, sport-related concussion, and varsity sport-related concussion. With increasing somatization the tendency to express psychosocial or emotional problems as physical ailments increases (American Psychiatric Association, 2013). Increased pre-concussion somatization symptom score has been shown to increase recovery time post-concussion (Nelson et al., 2016). Specifically, pre-injury somatization increased acute post-concussion symptoms, extending symptom recovery (Nelson et al., 2016). Thus, increasing baseline somatization symptoms may influence concussion risk by exacerbating symptoms, facilitating diagnosis. Thus it is unclear if increasing somatization directly increases concussion risk or diagnosis via increased symptom experience or reporting. Given the extremely healthy cadet population, small increases in psychological symptoms, particularly somatization, increase concussion risk. While results should be interpreted cautiously due to their small effect size, the effect of anxiety and somatization symptoms remained significant in multivariate models.

### Limitations

The current study is not without limitation. Best efforts were made to collect every incident concussion. However, due to non-reporting, it is possible incident concussions were missed which may bias the results. It is thought that 30% of collegiate athletes do not report their concussion (LaRoche et al., 2016; Kerr et al., 2016), however it is unknown whether similar non-reporting rates exist among cadets. Certain cadet characteristics, for example aspiring Air Force pilots, have lower reporting behaviors (D'Lauro et al., 2017). Thus, it is possible that certain cadet populations are at greater risk than as indicated in the current analysis. Thus, significant unreported concussions would underestimate the true concussion risk. However, more detailed estimates of cadet non-reporting and patterns among cadets are needed to further specify the direction of the bias.

Furthermore, given missing concussion data across sport levels, it is possible that the effects of varsity and intramural sport level are underestimated. For example, the magnitude of the varsity sport level effect may increase across concussion etiologies, increasing the effect across all concussion and within sport-related concussion. Moreover, the effect of varsity sport level within academy training-related concussions might become non-significant. While missing data may create biases, we expect the overall effect to be relatively small and not change the overall conclusions of the current study.

While the current study improved on the absolute number of females enrolled in a concussion study, the proportion of females to males remains unbalanced; however, this proportion is representative of the cadet population at the Service Academies (O'Connor et al., 2018). Caution should be used when generalizing results to non-cadet females or males. The current study is limited to risk factors associated with the presence or absence of a concussion. Rates, or time to injury, cannot be extrapolated. Additionally, while consistent predictors like sex and previous concussion were highly significant across all concussion settings, caution should be used when interpreting other predictors that are marginally significant (e.g. BSI Somatization) due to multiple comparisons.

## Conclusion

The current study demonstrated that concussion risk factors varied by concussion setting, highlighting unique and new at-risk populations. Female sex and previous concussion were the most consistent risk factors for concussion across all settings. Previously unidentified risk factors including freshman status and pre-injury somatization symptoms also significantly influence concussion risk among Service Academy cadets. By characterizing those at risk, better-targeted screening protocols can be developed to diagnose and treat concussion.

## Additional file

Additional file 1: **Table S1.** Univariate Associations with Sex. **Table S2.** Univariate Associations with Any Concussion. **Table S3.** Mixed Model Selection – Any Concussion. **Table S4.** Mixed Model Results – Any Concussion. **Table S5.** Univariate Associations with Any Sport-Related Concussion. **Table S6.** Mixed Model Selection – Any Sport-Related Concussion. **Table S7.** Mixed Model Results – Sport-Related Concussion. **Table S8.** Univariate Association with Any Academy Training-Related Concussion. **Table S9.** Mixed Model Selection – Any Academy Training-Related Concussion. **Table S10.** Mixed Model Results – Academy Training-Related Concussion. **Table S11.** Univariate Association with Free Time-Related Concussions. **Table S12.** Mixed Model Selection – Any Free Time-Related Concussion. **Table S13.** Mixed Model Results – Free Time-Related Concussion. **Table S14.** Mixed Model Selection – Any Concussion within Varsity Cadets. **Table S15.** Mixed Model Results – Any Concussion among Varsity Athlete Cadets. **Table S16.** Mixed Model Selection – Any Sport-Related Concussion within Varsity Cadets. **Table S17.** Mixed Model Results – Any Sport Concussion among Varsity Athletes. **Table S18.** Mixed Model Selection – Any Academy Training-Related Concussion within Varsity Cadets. **Table S19.** Mixed Model Selection – Any Free Time-Related Concussion within Varsity Cadets. **Table S20.** Description of risk factor variables and measures. (DOCX 94 kb)

## Abbreviations

BSI-18: Brief Symptom Inventory
CARE: Concussion Assessment, Research and Education
CI: Confidence Interval
SCAT: Sport Concussion Assessment Tool; OR: Odds Ratio
