# Supplementary material for: A cohort study to identify and evaluate concussion risk factors across multiple injury settings: findings from the CARE Consortium
Source: Inj Epidemiol. 2019 Jan 14;6:1. doi: 10.1186/s40621-018-0178-3 (PMC6330552; doi:10.1186/s40621-018-0178-3)
Supplement: Supplementary file 1 — Table S1. Univariate Associations with Sex. Table S2. Univariate Associations with Any Concussion. Table S3. Mixed Model Selection – Any Concussion. Table S4. Mixed Model Results – Any Concussion. Table S5. Univariate Associations with Any Sport-Related Concussion. Table S6. Mixed Model Selection – Any Sport-Related Concussion. Table S7. Mixed Model Results – Sport-Related Concussion. Table S8. Univariate Association with Any Academy Training-Related Concussion. Table S9. Mixed Model Selection – Any Academy Training-Related Concussion. Table S10. Mixed Model Results – Academy Training-Related Concussion. Table S11. Univariate Association with Free Time-Related Concussions. Table S12. Mixed Model Selection – Any Free Time-Related Concussion. Table S13. Mixed Model Results – Free Time-Related Concussion. Table S14. Mixed Model Selection – Any Concussion within Varsity Cadets. Table S15. Mixed Model Results – Any Concussion among Varsity Athlete Cadets. Table S16. Mixed Model Selection – Any Sport-Related Concussion within Varsity Cadets. Table S17. Mixed Model Results – Any Sport Concussion among Varsity Athletes. Table S18. Mixed Model Selection – Any Academy Training-Related Concussion within Varsity Cadets. Table S19. Mixed Model Selection – Any Free Time-Related Concussion within Varsity Cadets. Table S20. Description of risk factor variables and measures. (DOCX 94 kb) [file 40621_2018_178_MOESM1_ESM.docx]

Additional file 1: Table 1. Univariate Associations with Sex

|  | **Female** | **Male** | **Test** | **Effect Size** |
| --- | --- | --- | --- | --- |
| Sport Level |  |  |  |  |
| Varsity | 28.31% (830) | 71.69% (2102) | X^2^(2) = 118.14; p < 0.001 | Φ = 0.11 |
| Club | 29.58% (551) | 70.42% (1312) |  |  |
| Intramural | 19.61% (1045) | 80.39% (4285) |  |  |
| Varsity Contact Level^ |  |  |  |  |
| Contact | 20.08% (359) | 79.92% (1429) | X^2^(2) = 138.14; p < 0.001 | Φ = 0.22 |
| Limited-Contact | 41.62% (154) | 58.38% (216) |  |  |
| Non-Contact | 39.64% (283) | 60.36% (431) |  |  |
| Freshman |  |  |  |  |
| Yes | 26.67% (735) | 73.33% (2021) | X^2^(1) = 14.80; p < 0. 001 | d = 0.11 |
| No | 23.00% (1694) | 77.00% (5671) | OR: 0.1.22 (95% CI: 1.10-1.35) |  |
| Site |  |  |  |  |
| 1 | 20.46% (931) | 79.54% (3619) | X^2^(2) = 117.91; p < 0.001 | Φ = 0.11 |
| 2 | 24.84% (1162) | 75.16% (3516) |  |  |
| 3 | 36.94% (341) | 63.06% (582) |  |  |
| Previous Concussion |  |  |  |  |
| Yes | 21.20% (425) | 78.80% (1580) | X^2^(1) = 10.73; p < 0.01 | d = 0.11 |
| No | 24.69% (1982) | 75.31% (6046) | OR: 0.82 (95% CI: 0.73-0.92) |  |
| Headaches in past 3 months |  |  |  |  |
| Yes | 33.69% (756) | 66.31% (1488) | X^2^(1) = 146.93; p < 0.001 | d = 0.35 |
| No | 21.26% (1642) | 78.74% (6080) | OR: 1.88 (95% CI: 1.70-2.09) |  |
| Diagnosed Migraine Headache |  |  |  |  |
| Yes | 34.65% (88) | 65.35% (166) | X^2^(1) = 15.94; p < 0.001 | d = 0.30 |
| No | 23.80% (2317) | 76.20% (7420) | OR: 1.70 (95% CI: 1.31-2.21) |  |
| Diagnosed ADD/ADHD |  |  |  |  |

| Yes | 17.27% (24) | 82.73% (115) | X^2^(1) = 3.63; p = 0.06 | Φ = 0.02 |
| --- | --- | --- | --- | --- |
| No | 24.23% (2378) | 75.77% (7436) | OR: 0.65 (95% CI: 0.42-1.02) | d = 0.24 |
| Diagnosed Depression |  |  |  |  |
| Yes | 50.96% (53) | 49.04% (51) | X^2^(1) = 41.55; p < 0.001 | Φ = 0.06 |
| No | 23.79% (2353) | 76.21% (7536) | OR: 3.33 (95% CI: 2.26-4.90) | d = 0.66 |
| Brief Sensation Seeking Scale | 3.23 (0.67) | 3.46 (0.68) | T(9520) = -13.71; p < 0.001 | d = 0.32 |
| SCAT Symptoms |  |  |  |  |
| Number | 2.00 [0.00 – 5.00] | 1.00 [0.00 – 3.00] | Z = 13.19; p < 0.001¥ | d = 0.27 |
| Severity | 3.00 [0.00 – 8.00] | 1.00 [0.00 – 5.00] | Z = 13.28; p < 0.001 ¥ | d = 0.27 |
| BSI Total | 39.00 [33.00 - 47.00] | 36.00 [36.00 - 45.00] | Z= 14.04; p < 0.001 | d = 0.28 |
| Somatization | 41.00 [41.00 - 48.00] | 42.00 [42.00 - 42.00] | Z= 29.06; p < 0.001 | d = 0.61 |
| Depression | 40.00 [40.00 - 45.00] | 42.00 [42.00 - 45.00] | Z= 38.50; p < 0.001 | d = 0.83 |
| Anxiety | 38.00 [38.00 - 45.00] | 39.00 [39.00 - 39.00] | Z= 34.59; p < 0.001 | d = 0.74 |
| † Satterthwaite independent t-test | | | | |
| ¥ Wilcoxon two-sample test, medians and interquartile range reported  ^ Within Varsity level athletes only | | | | |

Additional file 1: Table 2. Univariate Associations with Any Concussion

|  | **Concussion** | **No Concussion** | **Test** | **Effect Size** |
| --- | --- | --- | --- | --- |
| Sport Level |  |  |  |  |
| Varsity | 9.14% (268) | 90.86% (2655) | *X*^2^(2) = 35.89; p <0.001 | Φ = 0.06 |
| Club | 7.09% (132) | 92.91% (1713) |  |  |
| Intramural | 5.65% (301) | 94.35% (5030) |  |  |
| Sex |  |  |  |  |
| Female | 11.30% (227) | 88.70% (2244) | *X*^2^(1) = 82.89; p <0.001 |  |
| Male | 5.84% (460) | 94.16% (7619) | OR: 2.04 (95% CI: 1.75-2.39) | d = 0.40 |
| Freshman |  |  |  |  |
| Yes | 13.38% (369) | 86.62% (2388) | *X*^2^(1) = 83.24; p <0.001 |  |
| No | 4.82% (355) | 94.18% (7011) | OR: 3.05 (95% CI: 2.62-3.56) | d = 0.62 |
| Site |  |  |  |  |
| 1 | 6.15% (280) | 93.85% (4301) | *X*^2^(2) = 58.35; p <0.001 | Φ = 0.08 |
| 2 | 9.00% (421) | 91.00% (4258) |  |  |
| 3 | 2.71% (25) | 97.29% (899) |  |  |
| Previous Concussion |  |  |  |  |
| Yes | 10.72% (215) | 89.28% (1790) | *X*^2^(1) = 57.92; p <0.001 |  |
| No | 5.92% (475) | 94.08% (7555) | OR: 1.91 (95% CI: 1.61-2.26) | d = 0.36 |
| Headaches in past 3 months |  |  |  |  |
| Yes | 11.18% (251) | 88.82% (1994) | *X*^2^(1) = 82.04; p <0.001 |  |
| No | 5.67% (438) | 94.33% (7285) | OR: 2.09 (95% CI: 1.78-2.46) | d = 0.41 |
| Diagnosed Migraine Headache |  |  |  |  |
| Yes | 11.37% (29) | 88.63% (226) | *X*^2^(1) = 8.22; p <0.01 |  |
| No | 6.77% (659) | 93.23% (9079) | OR: 1.77 (95% CI: 1.19-2.62) | d = 0.32 |
| Diagnosed ADD/ADHD |  |  |  |  |
| Yes | 12.95% (18) | 87.05% (130) | *X*^2^(1) = 8.33; p <0.01 |  |
| No | 6.73% (661) | 93.27% (9155) | OR: 2.06 (95% CI: 1.25-3.40) | d = 0.40 |
| Diagnosed Depression |  |  |  |  |
| Yes | 13.46% (14) | 86.54% (90) | *X*^2^(1) = 7.03; p <0.01 |  |
| No | 6.83% (676) | 93.17% (9215) | OR: 2.12 (95% CI: 1.20-3.74) | d = 0.42 |
| Brief Sensation Seeking Scale | 3.37 (0.66) | 3.40 (0.68) | t(9522) = 1.27; p =0.21 | d = 0.04 |
| Baseline SCAT Symptoms |  |  |  |  |
| Number | 2.00 [ 0.00-6.00] | 1.00 [0.00 - 3.00] | Z=8.79; p < 0.001^¥^ | d = 0.18 |
| Severity | 4.00 [0.00 - 11.00] | 1.00 [0.00 - 5.00] | Z=9.23; p < 0.001^¥^ | d = 0.19 |
| Baseline BSI Total | 39.00 [36.00 - 48.00] | 36.00 [36.00 - 45.00] | Z= 5.22; p < 0.001^¥^ | d = 0.10 |
| Somatization | 42.00 [42.00 - 50.00] | 42.00 [42.00 - 42.00] | Z= 5.73; p < 0.001^¥^ | d = 0.11 |
| Depression | 42.00 [42.00 - 45.00] | 42.00 [42.00 - 45.00] | Z= 0.18; p = 0.86^¥^ | d = 0.004 |
| Anxiety | 39.00 [39.00 - 47.00] | 39.00 [39.00 - 39.00] | Z= 1.36; p = 0.18^¥^ | d =0.03 |
| † Satterthwaite independent t-test  ¥ Wilcoxon two-sample test, medians and interquartile range reported | | | | |

Additional file 1: **Table 3**. Mixed Model Selection – Any Concussion

|  | Model 1 | Model 2 | Model 3 | Model 4 | Model 5 | Model 6 | Model 7 | Model 8 | Model 9 | Model 10 | Model 11 | Model 12 |
| --- | --- | --- | --- | --- | --- | --- | --- | --- | --- | --- | --- | --- |
| Sex | P < 0.0001 | P < 0.0001 | P < 0.0001 | P < 0.0001 | P < 0.0001 | P < 0.0001 | P < 0.0001 | P < 0.0001 | P < 0.0001 | P < 0.0001 | P < 0.0001 | P < 0.0001 |
| Sport Level |  | P < 0.0001 | P < 0.0001 | P < 0.0001 | P < 0.0001 | P < 0.0001 | P < 0.0001 | P < 0.0001 | P < 0.0001 | P < 0.0001 | P < 0.0001 | P < 0.0001 |
| Freshman |  |  | P < 0.0001 | P < 0.0001 | P < 0.0001 | P < 0.0001 | P < 0.0001 | P < 0.0001 | P < 0.0001 | P < 0.0001 | P < 0.0001 | P < 0.0001 |
| Previous Concussion |  |  |  | P < 0.0001 | P < 0.0001 | P < 0.0001 | P < 0.0001 | P < 0.0001 | P < 0.0001 | P < 0.0001 | P < 0.0001 | P < 0.0001 |
| Headache in the past three months |  |  |  |  | P < 0.0001 | P < 0.0001 | P < 0.0001 | P < 0.0001 | P < 0.0001 | P < 0.0001 | P < 0.0001 | P < 0.0001 |
| Migraine Headache |  |  |  |  |  | p = 0.22 |  |  |  |  |  |  |
| ADD/ADHD |  |  |  |  |  |  | p < 0.01 | p = 0.01 | p < 0.01 | p < 0.01 | p < 0.01 | p < 0.01 |
| Depression |  |  |  |  |  |  |  | p = 0.10 |  |  |  |  |
| BSI Somatization |  |  |  |  |  |  |  |  | p < 0.001 | p < 0.001 | p = 0.08 | p = 0.02 |
| BSI Depression |  |  |  |  |  |  |  |  | p = 0.30 |  |  |  |
| BSI Anxiety |  |  |  |  |  |  |  |  | p = 0.38 |  |  |  |
| SCAT Severity Score |  |  |  |  |  |  |  |  |  |  | p = 0.18 |  |
| BSSS |  |  |  |  |  |  |  |  |  |  |  | p = 0.95 |
| -2logLikelihood | 4755.67 | 4714.51 | 4527.09 | 4468.74 | 4441.88 | 4440.47 | 4435.24 | 4432.82 | 4397.95 | 4406.3 | 4399.13 | 4252.05 |
| BIC | 4758.96 | 4720 | 4533.68 | 4476.43 | 4450.67 | 4450.36 | 4445.13 | 4443.81 | 4411.14 | 4417.29 | 4411.21 | 4264.14 |

Additional file 1: Table 4. Mixed Model Results – Any Concussion

| **Parameter** | **Level** | **Estimate** | **Standard Error** | **t Value** | **Pr > \|t\|** | **Odds Ratio** | **Lower** | **Upper** |
| --- | --- | --- | --- | --- | --- | --- | --- | --- |
| Intercept |  | -5.09 | 0.58 | -8.80 | 0.01 |  |  |  |
| Sex | Female | 0.70 | 0.09 | 8.01 | <.0001 | 2.02 | 1.70 | 2.40 |
|  | Male | Ref. |  |  |  |  |  |  |
| Sport Level | Varsity | 0.48 | 0.10 | 5.02 | <.0001 | 1.61 | 1.34 | 1.95 |
|  | Club | 0.31 | 0.11 | 2.71 | 0.01 | 1.36 | 1.09 | 1.70 |
|  | Intramural | Ref. |  |  |  |  |  |  |
| Freshmen | Yes | 1.00 | 0.09 | 11.10 | <.0001 | 2.73 | 2.29 | 3.26 |
|  | No | Ref. |  |  |  |  |  |  |
| Previous Concussion | Yes | 0.68 | 0.09 | 7.40 | <.0001 | 1.98 | 1.65 | 2.37 |
|  | No | Ref. |  |  |  |  |  |  |
| Headache in the past three months | Yes | 0.40 | 0.09 | 4.37 | <.0001 | 1.50 | 1.25 | 1.80 |
|  | No | Ref. |  |  |  |  |  |  |
| ADD/ADHD | Yes | 0.73 | 0.27 | 2.73 | 0.01 | 2.08 | 1.23 | 3.52 |
|  | No | Ref. |  |  |  |  |  |  |
| BSI Somatization |  | 0.02 | 0.01 | 3.36 | 0.00 | 1.02 | 1.01 | 1.03 |
| Odds ratios for categorical predictors estimated at BSI somatization mean (44.57) | | | | | | | | |

Additional file 1: Table 5. Univariate Associations with Any Sport-Related Concussion

|  | **Concussion** | **No Concussion** | **Test** | **Effect Size** |
| --- | --- | --- | --- | --- |
| Sport Level |  |  |  |  |
| Varsity | 5.81% (169) | 94.19% (2739) | *X*^2^(2) = 140.82; p <0.001 | Φ = 0.11 |
| Club | 4.25% (79) | 95.75% (1779) |  |  |
| Intramural | 1.23% (65) | 98.77% (5233) |  |  |
| Sex |  |  |  |  |
| Female | 4.36% (105) | 95.64% (2302) | *X*^2^(1) = 14.00; p <0.001 | d = 0.25 |
| Male | 2.83% (217) | 97.17% (7463) | OR: 1.57 (95% CI: 1.24-1.99) |  |
| Contact Level^ |  |  |  |  |
| Contact | 8.12% (144) | 91.88% (1630) | *X*^2^(2) = 42.40; p <0.001 | Φ = 1.22 |
| Limited-Contact | 3.83% (14) | 96.17% (352) |  |  |
| Non-Contact | 1.55% (11) | 98.45% (697) |  |  |
| Freshman |  |  |  |  |
| Yes | 5.02% (137) | 94.98% (2594) | *X*^2^(1) = 40.43; p <0.001 | d = 0.40 |
| No | 2.51% (184) | 97.49% (7144) | OR: 2.05 (95% CI: 1.64-2.57) |  |
| Site |  |  |  |  |
| 1 | 3.31% (150) | 96.69% (4380) | *X*^2^(2) = 11.81; p <0.01 | Φ = 0.03 |
| 2 | 3.45% (160) | 96.55% (4479) |  |  |
| 3 | 1.30% (12) | 98.70% (908) |  |  |
| Previous Concussion |  |  |  |  |
| Yes | 5.39% (107) | 94.61% (1880) | *X*^2^(1) = 44.27; p <0.001 | d = 0.44 |
| No | 2.50% (200) | 97.50% (7787) | OR: 2.21 (95% CI: 1.74-2.82) |  |
| Headache in the past three months |  |  |  |  |
| Yes | 4.64% (103) | 95.36% (2116) | *X*^2^(1) = 23.04; p <0.001 | d = 0.32 |
| No | 2.64% (203) | 97.36% (7485) | OR: 1.79 (95% CI: 1.41-2.29) |  |
| Diagnosed Migraine Headache |  |  |  |  |
| Yes | 5.16% (13) | 94.84% (239) | *X*^2^(1) = 3.79; p = 0.05 |  |
| No | 3.02% (292) | 96.98% (9387) | OR: 1.75 (95% CI: 0.99-3.09) |  |
| Diagnosed ADD/ADHD |  |  |  |  |
| Yes | 3.65% (5) | 96.35% (132) | *X*^2^(1) = 0.16; p = 0.62 |  |
| No | 3.06% (299) | 96.94% (9466) | OR: 1.20 (95% CI: 0.49-2.95) |  |
| Diagnosed Depression |  |  |  |  |
| Yes | 3.88% (4) | 96.12% (99) | *X*^2^(1) = 0.22; p = 0.56 |  |
| No | 3.07% (302) | 96.93% (9528) | OR: 1.27 (95% CI: 0.47-3.49) |  |
| Brief Sensation Seeking Scale | 3.38 (0.69) | 3.40 (0.68) | t(9478) = 0.48; p =0.63 |  |
| SCAT Symptoms |  |  |  |  |
| Number | 2.00 [0.00 - 5.00] | 1.00 [0.00 - 4.00] | Z=3.19; p < 0.001^¥^ | d = 0.06 |
| Severity | 2.00 [0.00 - 9.00] | 2.00 [0.00 - 6.00] | Z=3.46; p < 0.001^¥^ | d = 0.07 |
| BSI Total | 36.00 [36.00 - 47.00] | 36.00 [36.00 - 45.00] | Z= 2.24; p = 0.02^¥^ | d = 0.05 |
| Somatization | 42.00 [42.00 - 48.00] | 42.00 [42.00 - 42.00] | Z= 2.73; p < 0.01^¥^ | d = 0.06 |
| Depression | 42.00 [42.00 - 45.00] | 42.00 [42.00 - 45.00] | Z= -0.38; p = 0.70^¥^ |  |
| Anxiety | 39.00 [39.00 - 47.00] | 39.00 [39.00 - 39.00] | Z= 0.58; p = 0.56^¥^ |  |
| † Satterthwaite independent t-test  ¥ Wilcoxon two-sample test, medians and interquartile range reported  ^ Only Varsity cadets  *** p < 0.001, ** p < 0.01, * p < 0.05 | | | | |

Additional file 1: **Table 6**. Mixed Model Selection – Any Sport-Related Concussion

|  | Model 1 | Model 2 | Model 3 | Model 4 | Model 5 | Model 6 | Model 7 | Model 8 | Model 9 | Model 10 | Model 11 | Model 12 |
| --- | --- | --- | --- | --- | --- | --- | --- | --- | --- | --- | --- | --- |
| Sex | p < 0.0001 | p < 0.01 | p < 0.01 | p < 0.01 | p < 0.01 | p < 0.01 | p < 0.01 | p < 0.01 | p < 0.01 | p = 0.01 | p = 0.01 | p < 0.01 |
| Sport Level |  | p < 0.0001 | p < 0.0001 | p < 0.0001 | p < 0.0001 | p < 0.0001 | p < 0.0001 | p < 0.0001 | p < 0.0001 | p < 0.0001 | p < 0.0001 | p < 0.0001 |
| Freshman |  |  | p < 0.0001 | p < 0.0001 | p < 0.0001 | p < 0.0001 | p < 0.0001 | p < 0.0001 | p < 0.0001 | p < 0.001 | p < 0.001 | p < 0.01 |
| Previous Concussion |  |  |  | p < 0.0001 | p < 0.0001 | p < 0.0001 | p < 0.0001 | p < 0.0001 | p < 0.0001 | p < 0.0001 | p < 0.0001 | p < 0.0001 |
| Headache in the past three months |  |  |  |  | p < 0.0001 | p < 0.0001 | p < 0.0001 | p < 0.0001 | p < 0.0001 | p < 0.01 | p < 0.01 | p < 0.01 |
| Migraine Headache |  |  |  |  |  | p = 0.26 |  |  |  |  |  |  |
| ADD/ADHD |  |  |  |  |  |  | p = 0.73 |  |  |  |  |  |
| Depression |  |  |  |  |  |  |  | p = 0.56 |  |  |  |  |
| BSI Somatization |  |  |  |  |  |  |  |  | p=0.02 | p < 0.01 | p = 0.11 | p = 0.01 |
| BSI Depression |  |  |  |  |  |  |  |  | p = 0.86 |  |  |  |
| BSI Anxiety |  |  |  |  |  |  |  |  | p = 0.66 |  |  |  |
| SCAT Severity Score |  |  |  |  |  |  |  |  |  |  | p = 0.51 |  |
| BSSS |  |  |  |  |  |  |  |  |  |  |  | p = 0.69 |
| -2logLikelihood | 2670.75 | 2515.52 | 2490.17 | 2459.11 | 2444.97 | 2443.8 | 2444.86 | 2444.67 | 2422.16 | 2422.59 | 2416.12 | 2358.29 |
| BIC | 2674.05 | 2521.01 | 2496.76 | 2466.8 | 2453.76 | 2453.69 | 2454.75 | 2454.55 | 2434.25 | 2432.48 | 2427.1 | 2369.27 |

Additional file 1: Table 7. Mixed Model Results – Sport-Related Concussion

| **Parameter** | **Level** | **Estimate** | **Standard Error** | **t Value** | **Pr > \|t\|** | **Odds Ratio** | **Lower** | **Upper** |
| --- | --- | --- | --- | --- | --- | --- | --- | --- |
| Intercept |  | -6.54 | 0.69 | -9.46 | 0.01 |  |  |  |
| Sex | Female | 0.32 | 0.13 | 2.45 | 0.01 | 1.38 | 1.07 | 1.78 |
|  | Male | Ref. |  |  |  |  |  |  |
| Sport Level | Varsity | 1.67 | 0.16 | 10.78 | <.0001 | 5.33 | 3.93 | 7.22 |
|  | Club | 1.35 | 0.17 | 7.75 | <.0001 | 3.87 | 2.75 | 5.45 |
|  | Intramural | Ref. |  |  |  |  |  |  |
| Freshmen | Yes | 0.47 | 0.13 | 3.57 | 0.00 | 1.60 | 1.24 | 2.06 |
|  | No | Ref. |  |  |  |  |  |  |
| Previous Concussion | Yes | 0.70 | 0.13 | 5.44 | <.0001 | 2.01 | 1.56 | 2.58 |
|  | No | Ref. |  |  |  |  |  |  |
| Headache in the past three months | Yes | 0.43 | 0.14 | 3.19 | 0.00 | 1.54 | 1.18 | 2.01 |
|  | No | Ref. |  |  |  |  |  |  |
| BSI Somatization |  | 0.02 | 0.01 | 2.69 | 0.01 | 1.03 | 1.01 | 1.04 |
| Odds ratios estimated at mean BSI Somatization symptom score (44.58)  Effect of BSI Somatization symptom score is assessed as one unit offsets from the mean. | | | | | | | | |

Additional file 1: Table 8. Univariate Association with Any Academy Training-Related Concussion

|  | **Concussion** | **No Concussion** | **Test** | **Effect Size** |
| --- | --- | --- | --- | --- |
| Sport Level |  |  |  |  |
| Varsity | 1.82% (53) | 98.18% (2855) | *X*^2^(2) = 10.07; p <0.01 | Φ = 0.03 |
| Club | 1.99% (37) | 98.01% (1821) |  |  |
| Intramural | 2.85% (151) | 97.15% (5147) |  |  |
| Sex |  |  |  |  |
| Female | 3.91% (94) | 96.09% (2313) | *X*^2^(1) = 29.59; p <0.001 | d = 0.39 |
| Male | 1.95% (150) | 98.05% (7530) | OR: 2.04 (95% CI: 1.57-2.65) |  |
| Freshman |  |  |  |  |
| Yes | 6.77% (185) | 93.23% (2546) | *X*^2^(1) = 302.06; p <0.001 | d = 1.22 |
| No | 0.79% (58) | 99.21% (7270) | OR: 9.11 (95% CI: 6.76-12.27) |  |
| Site |  |  |  |  |
| 1 | 1.66% (75) | 98.34% (4455) | *X*^2^(2) = 57.49; p <0.001 | Φ = 0.08 |
| 2 | 3.60% (167) | 96.40% (4472) |  |  |
| 3 | 0.22% (2) | 99.78% (918) |  |  |
| Previous Concussion |  |  |  |  |
| Yes | 3.37% (67) | 96.63% (1920) | *X*^2^(1) = 1035; p <0.01 | d = 0.26 |
| No | 2.14% (171) | 97.86% (7816) | OR: 1.59 (95% CI: 1.20-2.12) |  |
| Headache in the past three months |  |  |  |  |
| Yes | 4.64% (103) | 95.36% (2116) | *X*^2^(1) = 60.36; p <0.001 | d = 0.55 |
| No | 1.77% (103) | 98.23% (7552) | OR: 2.70 (95% CI: 2.08-3.51) |  |
| Diagnosed Migraine Headache |  |  |  |  |
| Yes | 3.97% (10) | 96.03% (242) | *X*^2^(1) = 2.68; p = 0.10 |  |
| No | 2.37% (229) | 97.63% (9450) | OR: 1.71 (95% CI: 0.89-3.25) |  |
| Diagnosed ADD/ADHD |  |  |  |  |
| Yes | 7.30% (10) | 92.70 (127) | *X*^2^(1) = 13.96; p <0.01 | d = 0.65 |
| No | 2.36% (230) | 97.64% (9535) | OR: 3.26 (95% CI: 1.69-6.30) |  |
| Diagnosed Depression |  |  |  |  |
| Yes | 5.83% (6) | 94.17% (97) | *X*^2^(1) = 5.13; p = 0.04 | d = 0.51 |
| No | 2.38% (234) | 97.62% (9596) | OR: 2.53 (95% CI: 1.10-5.84) |  |
| Brief Sensation Seeking Scale | 3.35 (0.57) | 3.40 (0.69) | t(252.31) = 1.48; p =0.14^†^ |  |
| SCAT Symptoms |  |  |  |  |
| Number | 4.00 [1.00 - 9.50] | 1.00 [0.00 - 4.00] | Z=10.16; p < 0.001^¥^ | d = 0.20 |
| Severity | 7.00 [1.50 - 18.00] | 1.00 [0.00 - 5.00] | Z= 10.42; p < 0.001^¥^ | d = 0.21 |
| BSI Total | 45.00 [36.00 - 52.00] | 45.00 [36.00 - 52.00] | Z= 8.17; p < 0.001^¥^ | d = 0.16 |
| Somatization | 48.00 [42.00 - 58.00] | 48.00 [42.00 - 58.00] | Z= 7.97; p < 0.001^¥^ | d = 0.16 |
| Depression | 42.00 [42.00 - 48.00] | 42.00 [42.00 - 48.00] | Z= 3.98; p < 0.001^¥^ | d = 0.08 |
| Anxiety | 39.00 [39.00 - 48.00] | 39.00 [39.00 - 48.00] | Z= 4.67; p < 0.001^¥^ | d = 0.09 |
| † Satterthwaite independent t-test  ¥ Wilcoxon two-sample test, medians and interquartile range reported  *** p < 0.001, ** p < 0.01, * p < 0.05 | | | | |

Additional file 1: **Table 9**. Mixed Model Selection – Any Academy Training-Related Concussion

|  | Model 1 | Model 2 | Model 3 | Model 4 | Model 5 | Model 6 | Model 7 | Model 8 | Model 9 | Model 10 | Model 11 | Model 12 |
| --- | --- | --- | --- | --- | --- | --- | --- | --- | --- | --- | --- | --- |
| Sex | p < 0.0001 | p < 0.0001 | p < 0.0001 | p < 0.0001 | p < 0.0001 | p < 0.0001 | p < 0.0001 | p < 0.0001 | p < 0.0001 | p < 0.001 | p < 0.001 | p < 0.001 |
| Sport Level |  | p = 0.06 | p < 0.01 | p < 0.01 | p < 0.01 | p < 0.01 | p < 0.01 | p < 0.01 | p < 0.01 | p < 0.01 | p < 0.01 | p < 0.01 |
| Freshman |  |  | p < 0.0001 | p < 0.0001 | p < 0.0001 | p < 0.0001 | p < 0.0001 | p < 0.0001 | p < 0.0001 | p < 0.0001 | p < 0.0001 | p < 0.0001 |
| Previous Concussion |  |  |  | p < 0.0001 | p < 0.001 | p < 0.001 | p < 0.001 | p < 0.001 | p < 0.001 | p < 0.001 | p < 0.001 | p < 0.001 |
| Headache in the past three months |  |  |  |  | p < 0.001 | p < 0.001 | p < 0.001 | p < 0.001 | p < 0.01 | p < 0.01 | p < 0.01 | p < 0.01 |
| Migraine Headache |  |  |  |  |  | p = 0.86 |  |  |  |  |  |  |
| ADD/ADHD |  |  |  |  |  |  | p < 0.001 | p < 0.01 | p < 0.001 | p < 0.001 | p < 0.001 | p < 0.001 |
| Depression |  |  |  |  |  |  |  | p = 0.22 |  |  |  |  |
| BSI Somatization |  |  |  |  |  |  |  |  | p < 0.01 | p = 0.02 | p = 0.35 | p = 0.03 |
| Sex*Somatization |  |  |  |  |  |  |  |  | p < 0.01 | p = 0.01 | p = 0.02 | p = 0.01 |
| BSI Depression |  |  |  |  |  |  |  |  | p = 0.29 |  |  |  |
| BSI Anxiety |  |  |  |  |  |  |  |  | p = 0.42 |  |  |  |
| SCAT Severity Score |  |  |  |  |  |  |  |  |  |  | p = 0.18 |  |
| BSSS |  |  |  |  |  |  |  |  |  |  |  | p = 0.77 |
| -2logLikelihood | 2133.19 | 2127.25 | 1867.21 | 1852.2 | 1838.73 | 1838.7 | 1828.64 | 1827.29 | 1802.9 | 1809.54 | 1800.88 | 1771.62 |
| BIC | 2136.49 | 2132.74 | 1873.8 | 1859.89 | 1847.52 | 1848.58 | 1838.53 | 1838.28 | 1817.18 | 1821.63 | 1814.07 | 1784.8 |

Additional file 1: Table 10. Mixed Model Results – Academy Training-Related Concussion

| **Parameter** | **Level** | **Estimate** | **Standard Error** | **t Value** | **Pr > \|t\|** | **Odds Ratio** | **Lower** | **Upper** |  |
| --- | --- | --- | --- | --- | --- | --- | --- | --- | --- |
| Intercept |  | -7.81 | 0.98 | -8.00 | 0.02 |  |  |  |  |
| Sex | Female | 2.69 | 0.79 | 3.43 | 0.00 | 2.32 | 1.70 | 3.18 |  |
|  | Male | Ref. |  |  |  |  |  |  |  |
| Sport Level | Varsity | -0.59 | 0.17 | -3.44 | 0.00 | 0.56 | 0.40 | 0.78 |  |
|  | Club | -0.28 | 0.20 | -1.42 | 0.15 | 0.76 | 0.52 | 1.11 |  |
|  | Intramurals | Ref. |  |  |  |  |  |  |  |
| Freshmen | Yes | 2.10 | 0.17 | 12.47 | <.0001 | 8.17 | 5.87 | 11.37 |  |
|  | No | Ref. |  |  |  |  |  |  |  |
| Previous Concussion | Yes | 0.55 | 0.16 | 3.49 | 0.00 | 1.73 | 1.27 | 2.36 |  |
|  | No | Ref. |  |  |  |  |  |  |  |
| Headache in the past three months | Yes | 0.42 | 0.15 | 2.88 | 0.00 | 1.53 | 1.15 | 2.04 |  |
|  | No | Ref. |  |  |  |  |  |  |  |
| ADD/ADHD | Yes | 1.27 | 0.37 | 3.44 | 0.00 | 3.55 | 1.73 | 7.32 |  |
|  | No | Ref. |  |  |  |  |  |  |  |
| BSI Somatization |  | 0.04 | 0.01 | 3.81 | 0.00 | 1.04 | 1.02 | 1.07 |  |
| Sex*BSI Somatization | Female | -0.04 | 0.02 | -2.64 | 0.01 | 0.96 | 0.93 | 0.99 |  |
| Odds ratios estimated at mean BSI Somatization symptom score (44.57)  Effect of BSI Somatization symptom score is assessed as one unit offsets from the mean. | | | | | | | | | |

Additional file 1: Table 11. Univariate Association with Free Time-Related Concussions

|  | **Concussion** | **No Concussion** | **Test** | **Effect Size** |
| --- | --- | --- | --- | --- |
| Sport Level |  |  |  |  |
| Varsity | 0.96% (28) | 99.04% (2880) | *X*^2^(2) = 0.41; p = 0.81 |  |
| Club | 0.97% (18) | 99.03% (1840) |  |  |
| Intramural | 1.09% (58) | 98.91% (5240) |  |  |
| Sex |  |  |  |  |
| Female | 2.24% (54) | 97.76% (2353) | *X*^2^(1) = 33.25; p <0.001 | d = 0.57 |
| Male | 0.81% (62) | 99.19% (7618) | OR: 2.82 (95% CI: 1.95-4.07) |  |
| Freshman |  |  |  |  |
| Yes | 1.43% (39) | 98.57% (2692) | *X*^2^(1) = 2.48; p = 0.12 |  |
| No | 1.05% (39) | 98.95% (7251) | OR: 1.36 (95% CI: 0.92-2.01) |  |
| Site |  |  |  |  |
| 1 | 0.95% (43) | 99.05% (4487) | *X*^2^(2) = 4.82; p = 0.09 |  |
| 2 | 1.40% (65) | 98.60% (4574) |  |  |
| 3 | 0.87% (8) | 99.13% (912) |  |  |
| Previous Concussion |  |  |  |  |
| Yes | 1.46% (29) | 98.54% (1958) | *X*^2^(1) = 4.18; p = 0.04 | d = 0.25 |
| No | 0.94% (75) | 99.06% (7912) | OR: 1.56 (95% CI: 1.01-2.41) |  |
| Headache in the past three months |  |  |  |  |
| Yes | 1.31% (29) | 98.69% (2190) | *X*^2^(1) = 1.98; p = 0.16 |  |
| No | 0.96% (74) | 99.04% (7614) | OR: 1.36 (95% CI: 0.88-2.10) |  |
| Diagnosed Migraine Headache |  |  |  |  |
| Yes | 1.98% (5) | 98.02% (247) | *X*^2^(1) = 2.33; p = 0.19 |  |
| No | 1.00% (97) | 99.00% (9582) | OR: 1.99 (95% CI: 0.81-4.96) |  |
| Diagnosed ADD/ADHD |  |  |  |  |
| Yes | 1.46% (2) | 98.54% (135) | *X*^2^(1) = 0.25; p = 0.65 |  |
| No | 1.02% (100) | 98.98% (9655) | OR: 1.43 (95% CI: 0.35-5.87) |  |
| Diagnosed Depression |  |  |  |  |
| Yes | 2.91% (3) | 97.09% (100) | *X*^2^(1) = 3.64; p = 0.09 |  |
| No | 1.01% (99) | 98.99% (9731) | OR: 2.95 (95% CI: 0.92-9.46) |  |
| Brief Sensation Seeking Scale | 3.46 (0.76) | 3.40 (0.68) | t(9478) = -0.84; p = 0.42 |  |
| SCAT Symptoms |  |  |  |  |
| Number | 2.00 [0.00 - 5.00] | 1.00 [0.00 - 4.00] | Z = 1.95; p = 0.5^¥^ |  |
| Severity | 3.00 [0.00 - 7.00] | 2.00 [0.00 - 6.00] | Z= 2.04; p = 0.04^¥^ | d = 0.04 |
| BSI Total | 36.00 [36.00 - 45.00] | 36.00 [36.00 - 45.00] | Z= -0.79; p = 0.43^¥^ |  |
| Somatization | 42.00 [41.00 - 48.00] | 42.00 [42.00 - 42.00] | Z= -1.28; p = 0.20^¥^ |  |
| Depression | 42.00 [40.00 -45.00] | 42.00 [42.00 - 45.00] | Z= -2.04; p = 0.04^¥^ | d = 0.04 |
| Anxiety | 39.00 [38.00 - 45.00] | 39.00 [39.00 - 39.00] | Z= -1.97; p = 0.05^¥^ |  |
| † Satterthwaite independent t-test  ¥ Wilcoxon two-sample test, medians and interquartile range reported  *** p < 0.001, ** p < 0.01, * p < 0.05 | | | | |

Additional file 1: **Table 12**. Mixed Model Selection – Any Free Time-Related Concussion

|  | Model 1 | Model 2 | Model 3 | Model 4 | Model 5 | Model 6 | Model 7 | Model 8 | Model 9 | Model 10 | Model 11 | Model 12 |
| --- | --- | --- | --- | --- | --- | --- | --- | --- | --- | --- | --- | --- |
| Sex | p < 0.0001 | p < 0.0001 | p < 0.0001 | p < 0.0001 | p < 0.0001 | p < 0.0001 | p < 0.0001 | p < 0.0001 | p < 0.0001 | p < 0.0001 | p < 0.0001 | p < 0.0001 |
| Sport Level |  | p = 0.63 |  |  |  |  |  |  |  |  |  |  |
| Freshman |  |  | p = 0.04 | p = 0.04 | p = 0.05 | p = 0.04 | p = 0.04 | p = 0.04 | p = 0.05 | p = 0.06 | p = 0.07 |  |
| Previous Concussion |  |  |  | p = 0.01 | p = 0.01 | p = 0.02 | p = 0.01 | p = 0.02 | p = 0.01 | p = 0.01 | p = 0.01 | p = 0.01 |
| Headache in the past three months |  |  |  |  | p = 0.83 |  |  |  |  |  |  |  |
| Migraine Headache |  |  |  |  |  | p = 0.33 |  |  |  |  |  |  |
| ADD/ADHD |  |  |  |  |  |  | p = 0.52 |  |  |  |  |  |
| Depression |  |  |  |  |  |  |  | p = 0.22 |  |  |  |  |
| BSI Somatization |  |  |  |  |  |  |  |  | p = 0.67 |  |  |  |
| BSI Depression |  |  |  |  |  |  |  |  | p = 0.40 |  |  |  |
| BSI Anxiety |  |  |  |  |  |  |  |  | p = 0.96 |  |  |  |
| SCAT Severity Score |  |  |  |  |  |  |  |  |  | p = 0.89 |  |  |
| BSSS |  |  |  |  |  |  |  |  |  |  | p = 0.06 | p = 0.06 |
| -2logLikelihood | 1101.1 | 1100.17 | 1097.21 | 1101.53 | 1091.49 | 1090.69 | 1091.18 | 1090.29 | 1089.81 | 1091.08 | 1067.76 | 1070.87 |
| BIC | 1104.39 | 1105.66 | 1101.6 | 1097.02 | 1098.08 | 1097.28 | 1097.77 | 1096.88 | 1098.6 | 1097.68 | 1074.35 | 1076.36 |

Additional file 1: Table 13. Mixed Model Results – Free Time-Related Concussion

| **Parameter** | **Level** | **Estimate** | **Standard Error** | **t Value** | **Pr > \|t\|** | **Odds Ratio** | **Lower** | **Upper** |
| --- | --- | --- | --- | --- | --- | --- | --- | --- |
| Intercept |  | -5.2944 | 0.2582 | -20.51 | 0.0024 |  |  |  |
| Sex | Female | 1.1167 | 0.201 | 5.56 | <.0001 | 3.055 | 2.06 | 4.529 |
|  | Male | Ref. |  |  |  |  |  |  |
| Freshmen | Yes | 0.4267 | 0.207 | 2.06 | 0.0393 | 1.532 | 1.021 | 2.299 |
|  | No | Ref. |  |  |  |  |  |  |
| Previous Concussion | Yes | 0.5516 | 0.2221 | 2.48 | 0.013 | 1.736 | 1.123 | 2.683 |
|  | No | Ref. |  |  |  |  |  |  |

Additional file 1: Table 14. Mixed Model Selection – Any Concussion within Varsity Cadets

|  | Model 1 | Model 2 | Model 3 | Model 4 | Model 5 | Model 6 | Model 7 | Model 8 | Model 9 | Model 10 | Model 11 | Model 12 |
| --- | --- | --- | --- | --- | --- | --- | --- | --- | --- | --- | --- | --- |
| Sex | p < 0.001 | p < 0.0001 | p < 0.0001 | p < 0.0001 | p < 0.0001 | p < 0.0001 | p < 0.0001 | p < 0.0001 | p < 0.01 | p < 0.01 | p < 0.01 | p < 0.001 |
| Contact Level |  | p < 0.0001 | p < 0.0001 | p < 0.001 | p < 0.001 | p < 0.001 | p < 0.001 | p < 0.001 | p < 0.0001 | p < 0.001 | p < 0.0001 | p < 0.001 |
| Freshman |  |  | p < 0.0001 | p < 0.0001 | p < 0.0001 | p < 0.0001 | p < 0.0001 | p < 0.0001 | p < 0.0001 | p < 0.0001 | p < 0.0001 | p < 0.0001 |
| Previous Concussion |  |  |  | p < 0.0001 | p < 0.001 | p < 0.001 | p < 0.001 | p < 0.001 | p < 0.001 | p < 0.001 | p < 0.001 | p < 0.001 |
| Headache in the past three months |  |  |  |  | p < 0.01 | p < 0.01 | p < 0.01 | p < 0.01 | p = 0.01 | p < 0.01 | p < 0.01 | p < 0.01 |
| Migraine Headache |  |  |  |  |  | p = 0.41 |  |  |  |  |  |  |
| ADD/ADHD |  |  |  |  |  |  | p = 0.97 |  |  |  |  |  |
| Depression |  |  |  |  |  |  |  | p = 0.87 |  |  |  |  |
| BSI Somatization |  |  |  |  |  |  |  |  | p = 0.06 |  |  |  |
| BSI Depression |  |  |  |  |  |  |  |  | p = 0.89 |  |  |  |
| BSI Anxiety |  |  |  |  |  |  |  |  | p = 0.42 | p = 0.97 | p = 0.45 | p = 0.90 |
| Sex*BSI Anxiety |  |  |  |  |  |  |  |  | p = 0.02 | p = 0.01 | p = 0.02 | p = 0.01 |
| SCAT Severity Score |  |  |  |  |  |  |  |  |  |  | p = 0.20 |  |
| BSSS |  |  |  |  |  |  |  |  |  |  |  | p = 0.82 |
| -2logLikelihood | 1566.5 | 1540.45 | 1506.14 | 1490.96 | 1482.23 | 1481.59 | 1482.23 | 1482.2 | 1469.62 | 1473.26 | 1471.65 | 1399.82 |
| BIC | 1569.8 | 1545.94 | 1512.73 | 1498.65 | 1491.02 | 1491.47 | 1492.11 | 1492.09 | 1482.81 | 1484.24 | 1483.74 | 1411.91 |

Additional file 1: Table 15. Mixed Model Results – Any Concussion among Varsity Athlete Cadets

| **Parameter** | **Level** | **Estimate** | **Standard Error** | **DF** | **t Value** | **Pr > \|t\|** | **Odds Ratio** | **Lower** | **Upper** |  |
| --- | --- | --- | --- | --- | --- | --- | --- | --- | --- | --- |
| Intercept |  | -5.36 | 0.80 | 2.00 | -6.70 | 0.02 |  |  |  |  |
| Sex | Female | 3.03 | 0.93 | 2696.00 | 3.25 | 0.00 | 2.11 | 1.56 | 2.86 |  |
|  | Male | Ref. |  |  |  |  |  |  |  |  |
| Contact Level | Contact | 0.89 | 0.21 | 2696.00 | 4.18 | <.0001 | 2.43 | 1.60 | 3.68 |  |
|  | Limited-Contact | 0.54 | 0.27 | 2696.00 | 1.97 | 0.05 | 1.72 | 1.00 | 2.93 |  |
|  | Non-Contact | Ref. |  |  |  |  |  |  |  |  |
| Freshmen | Yes | 0.77 | 0.15 | 2696.00 | 5.15 | <.0001 | 2.15 | 1.61 | 2.88 |  |
|  | No | Ref. |  |  |  |  |  |  |  |  |
| Previous Concussion | Yes | 0.58 | 0.15 | 2696.00 | 3.88 | 0.00 | 1.79 | 1.33 | 2.40 |  |
|  | No | Ref. |  |  |  |  |  |  |  |  |
| Headache in the past three months | Yes | 0.46 | 0.16 | 2696.00 | 2.88 | 0.00 | 1.58 | 1.16 | 2.16 |  |
|  | No | Ref. |  |  |  |  |  |  |  |  |
| BSI Anxiety |  | 0.03 | 0.01 | 2696.00 | 2.04 | 0.04 | 1.03 | 1.00 | 1.05 |  |
| Sex*BSI Anxiety | Female | -0.05 | 0.02 | 2696.00 | -2.49 | 0.01 | 0.95 | 0.91 | 0.99 |  |
| Odds ratios for categorical predictors estimated at mean of BSI Anxiety score (41.85)  Effect of BSI Anxiety score is assessed as one unit offsets from the mean. | | | | | | | | | | |

Additional file 1: **Table 16**. Mixed Model Selection – Any Sport-Related Concussion within Varsity Cadets

|  | Model 1 | Model 2 | Model 3 | Model 4 | Model 5 | Model 6 | Model 7 | Model 8 | Model 9 | Model 10 | Model 11 | Model 12 | Model 13 |
| --- | --- | --- | --- | --- | --- | --- | --- | --- | --- | --- | --- | --- | --- |
| Sex | p = 0.14 | p < 0.01 | p < 0.01 | p < 0.01 | p < 0.01 | p < 0.01 | p < 0.01 | p < 0.01 | p < 0.01 | p < 0.01 | p < 0.01 | p < 0.01 | p < 0.01 |
| Contact Level |  | p < 0.0001 | p < 0.0001 | p < 0.0001 | p < 0.0001 | p < 0.0001 | p < 0.0001 | p < 0.0001 | p < 0.0001 | p < 0.0001 | p < 0.0001 | p < 0.0001 | p < 0.0001 |
| Freshman |  |  | p = 0.03 | p = 0.02 | p = 0.04 | p = 0.04 | p = 0.04 | p = 0.04 | p = 0.21 | p = 0.20 |  |  |  |
| Previous Concussion |  |  |  | p < 0.001 | p < 0.01 | p < 0.01 | p < 0.01 | p < 0.01 | p < 0.01 | p < 0.01 | p < 0.01 | p < 0.01 | p < 0.001 |
| Headache in the past three months |  |  |  |  | p = 0.02 | p = 0.03 | p = 0.02 | p = 0.02 | p = 0.07 | p = 0.07 |  |  |  |
| Migraine Headache |  |  |  |  |  | p = 0.62 |  |  |  |  |  |  |  |
| ADD/ADHD |  |  |  |  |  |  | p = 0.77 |  |  |  |  |  |  |
| Depression |  |  |  |  |  |  |  | p = 0.98 |  |  |  |  |  |
| BSI Somatization |  |  |  |  |  |  |  |  | p = 0.09 | p = 0.03 | p < 0.001 | p = 0.08 | p < 0.01 |
| BSI Depression |  |  |  |  |  |  |  |  | p = 0.65 |  |  |  |  |
| BSI Anxiety |  |  |  |  |  |  |  |  | p = 0.71 |  |  |  |  |
| SCAT Severity Score |  |  |  |  |  |  |  |  |  |  |  | p = 0.39 |  |
| BSSS |  |  |  |  |  |  |  |  |  |  |  |  | p = 0.96 |
| -2logLikelihood | 1132.46 | 1080.23 | 1075.45 | 1064.72 | 1059.61 | 1059.39 | 1059.53 | 1059.61 | 1054.07 | 1054.33 | 1059.49 | 1058.73 | 1018.78 |
| BIC | 1135.75 | 1085.72 | 1082.04 | 1072.41 | 1068.4 | 1069.27 | 1069.42 | 1069.5 | 1066.15 | 1064.22 | 1067.18 | 1067.52 | 1027.56 |

Additional file 1: Table 17. Mixed Model Results – Any Sport Concussion among Varsity Athletes

| **Parameter** | **Level** | **Estimate** | **Standard** | **t Value** | **Pr > \|t\|** | **Odds Ratio** | **Lower** | **Upper** | **Effect Size** |  |
| --- | --- | --- | --- | --- | --- | --- | --- | --- | --- | --- |
| Intercept |  | -7.1173 | 0.9381 | -7.59 | 0.0169 |  |  |  |  |  |
| Sex | Female | 0.5378 | 0.1908 | 2.82 | 0.0049 | 1.712 | 1.178 | 2.489 | 0.297051 |  |
|  | Male | Ref. |  |  |  |  |  |  |  |  |
| Contact Level | Contact | 2.0257 | 0.3986 | 5.08 | <.0001 | 7.581 | 3.47 | 16.565 | 1.119141 |  |
|  | Limited-Contact | 1.2582 | 0.4763 | 2.64 | 0.0083 | 3.519 | 1.383 | 8.954 | 0.695125 |  |
|  | Non-Contact | Ref. |  |  |  |  |  |  |  |  |
| Previous Concussion | Yes | 0.5706 | 0.1773 | 3.22 | 0.0013 | 1.769 | 1.25 | 2.505 | 0.315146 |  |
|  | No | Ref. |  |  |  |  |  |  |  |  |
| BSI Somatization |  | 0.04098 | 0.01217 | 3.37 | 0.0008 | 1.042 | 1.017 | 1.067 | 0.02273 |  |
| Odds ratios for categorical predictors estimated at mean of BSI Somatization score (45.07)  Effect of BSI Somatization score is assessed as one unit offsets from the mean. | | | | | | | | | | |

Additional file 1: **Table 18**. Mixed Model Selection – Any Academy Training-Related Concussion within Varsity Cadets

|  | Model 1 | Model 2 | Model 3 | Model 4 | Model 5 | Model 6 | Model 7 | Model 8 | Model 9 | Model 10 | Model 11 |
| --- | --- | --- | --- | --- | --- | --- | --- | --- | --- | --- | --- |
| Sex | p = 0.06 | p = 0.07 | p = 0.04 | p = 0.03 | p = 0.03 | p = 0.03 | p = 0.03 | p = 0.04 | p = 0.09 | p = 0.03 | p = 0.05 |
| Contact Level |  | p = 0.83 | p < 0.0001 | |  |  |  |  |  |  |  |
| Freshman |  |  |  | p < 0.0001 | p < 0.0001 | p < 0.0001 | p < 0.0001 | p < 0.0001 | p < 0.0001 | p < 0.0001 | p < 0.0001 |
| Previous Concussion |  |  |  | p = 0.03 | p = 0.03 | p = 0.03 | p = 0.03 | p = 0.03 | p = 0.03 | p = 0.04 | p = 0.06 |
| Headache in the past three months |  |  |  |  | p = 0.68 |  |  |  |  |  |  |
| Migraine Headache |  |  |  |  |  | p = 0.89 |  |  |  |  |  |
| ADD/ADHD |  |  |  |  |  |  | p = 0.99 |  |  |  |  |
| Depression |  |  |  |  |  |  |  | p = 0.43 |  |  |  |
| BSI Somatization |  |  |  |  |  |  |  |  | p = 0.14 |  |  |
| BSI Depression |  |  |  |  |  |  |  |  | p= 0.25 |  |  |
| BSI Anxiety |  |  |  |  |  |  |  |  | p = 0.46 |  |  |
| SCAT Severity Score |  |  |  |  |  |  |  |  |  | p =0.14 |  |
| BSSS |  |  |  |  |  |  |  |  |  |  | p = 0.89 |
| -2logLikelihood | 490.58 | 490.22 | 424.64 | 420.35 | 420.18 | 420.33 | 418.48 | 419.83 | 415.4 | 417.48 | 408.59 |
| BIC | 493.88 | 495.72 | 429.04 | 425.84 | 426.77 | 426.92 | 425.07 | 426.42 | 424.19 | 424.08 | 415.18 |

Additional file 1: **Table 19**. Mixed Model Selection – Any Free Time-Related Concussion within Varsity Cadets

|  | Model 1 | Model 2 | Model 3 | Model 4 | Model 5 | Model 6 | Model 7 | Model 8 | Model 9 | Model 10 | Model 11 |
| --- | --- | --- | --- | --- | --- | --- | --- | --- | --- | --- | --- |
| Sex | p < 0.01 | p = 0.01 | p < 0.01 | p < 0.01 | p = 0.01 | p < 0.01 | p < 0.01 | p < 0.0001 | p < 0.001 | p = 0.01 | p < 0.01 |
| Contact Level |  | p = 0.85 |  |  |  |  |  |  |  |  |  |
| Freshman |  |  | p = 0.33 |  |  |  |  |  |  |  |  |
| Previous Concussion |  |  |  | p = 0.79 |  |  |  |  |  |  |  |
| Headache in the past three months |  |  |  |  | p = 0.42 |  |  |  |  |  |  |
| Migraine Headache |  |  |  |  |  | p = 0.24 |  |  |  |  |  |
| ADD/ADHD |  |  |  |  |  |  | p = 0.99 |  |  |  |  |
| Depression |  |  |  |  |  |  |  | p = 0.99 |  |  |  |
| BSI Somatization |  |  |  |  |  |  |  |  | p = 0.57 |  |  |
| BSI Depression |  |  |  |  |  |  |  |  | p = 0.47 |  |  |
| BSI Anxiety |  |  |  |  |  |  |  |  | p = 0.51 |  |  |
| SCAT Severity Score |  |  |  |  |  |  |  |  |  | p = 0.41 |  |
| BSSS |  |  |  |  |  |  |  |  |  |  | p = 0.58 |
| -2logLikelihood | 295.59 | 295.28 | 294.65 | 295.52 | 294.97 | 294.49 | 294.89 | 295.12 | 294.35 | 294.87 | 292.02 |
| BIC | 298.88 | 300.77 | 299.04 | 299.91 | 299.37 | 298.89 | 299.28 | 299.52 | 300.94 | 299.26 | 296.41 |

Additional file 1: **Table 20. Description of risk factor variables and measures**

| **Risk Factor Grouping** | **Measure/Variable** | **Description** |
| --- | --- | --- |
| Demographic | Sex | Variable collected on case report form |
| Medical/Well-Being | Anxiety Symptoms | Brief Symptom Inventory – 18 anxiety subscale |
|  | Depression Symptoms | Brief Symptom Inventory – 18 Depression subscale |
|  | Somatization Symptoms | Brief Symptom Inventory – 18 Somatization subscale |
|  | Previous Concussion | Variable collected on case report form |
|  | ADD/ADHD | Variable collected on case report form |
|  | Headache in the past three months | Variable collected on case report form |
|  | Migraine Headache | Variable collected on case report form |
|  | Concussion Symptoms | Standardized Concussion Assessment Tool – Symptom Inventory |
| Environment | Freshman | Variable collected on case report form |
|  | Sport Level | Variable collected on case report form |
|  | Contact Level | Variable collected on case report form |
|  | Site | The academy the cadet attended. Site was the random effect included in the mixed model. |
